# Xenotransplantation of Genetically Modified Neonatal Pig Islets Cures Diabetes in Baboons

**DOI:** 10.3389/fimmu.2022.898948

**Published:** 2022-06-16

**Authors:** Wayne J. Hawthorne, Evelyn J. Salvaris, Yi Vee Chew, Heather Burns, Joanne Hawkes, Helen Barlow, Min Hu, Andrew M. Lew, Mark B. Nottle, Philip J. O’Connell, Peter J. Cowan

**Affiliations:** ^1^ The Centre for Transplant & Renal Research, Westmead Institute for Medical Research, Westmead, NSW, Australia; ^2^ Department of Surgery, Westmead Hospital, School of Medical Sciences, University of Sydney, Westmead, NSW, Australia; ^3^ Immunology Research Centre, St. Vincent’s Hospital, Melbourne, VIC, Australia; ^4^ Division of Immunology, Walter and Eliza Hall Institute, Melbourne, VIC, Australia; ^5^ Department of Obstetrics and Gynaecology, University of Adelaide, Adelaide, SA, Australia; ^6^ Department of Medicine, University of Melbourne, Melbourne, VIC, Australia

**Keywords:** diabetes mellitus, insulin, hyperacute rejection (HAR), instant blood mediated inflammatory reaction (IBMIR), neonatal islet cell clusters, thrombosis, xenotransplantation

## Abstract

Xenotransplantation using porcine donors is rapidly approaching clinical applicability as an alternative therapy for treatment of many end-stage diseases including type 1 diabetes. Porcine neonatal islet cell clusters (NICC) have normalised blood sugar levels for relatively short periods in the preclinical diabetic rhesus model but have met with limited success in the stringent baboon model. Here we report that NICC from genetically modified (GM) pigs deleted for αGal and expressing the human complement regulators CD55 and CD59 can cure diabetes long-term in immunosuppressed baboons, with maximum graft survival exceeding 22 months. Five diabetic baboons were transplanted intraportally with 9,673 – 56,913 islet equivalents (IEQ) per kg recipient weight. Immunosuppression consisted of T cell depletion with an anti-CD2 mAb, tacrolimus for the first 4 months, and maintenance with belatacept and anti-CD154; no anti-inflammatory treatment or cytomegalovirus (CMV) prophylaxis/treatment was given. This protocol was well tolerated, with all recipients maintaining or gaining weight. Recipients became insulin-independent at a mean of 87 ± 43 days post-transplant and remained insulin-independent for 397 ± 174 days. Maximum graft survival was 675 days. Liver biopsies showed functional islets staining for all islet endocrine components, with no evidence of the inflammatory blood-mediated inflammatory reaction (IBMIR) and minimal leukocytic infiltration. The costimulation blockade-based immunosuppressive protocol prevented an anti-pig antibody response in all recipients. In conclusion, we demonstrate that genetic modification of the donor pig enables attenuation of early islet xenograft injury, and in conjunction with judicious immunosuppression provides excellent long-term function and graft survival in the diabetic baboon model.

## Introduction

An estimated 22 million people currently suffer from type 1 diabetes mellitus (1). This incredible burden is treated with various forms of exogenous insulin therapy but whole pancreas or islet cell transplantation remains the gold standard for treatment. Pancreas or islet transplantation restores glucose homeostasis without constant external monitoring and prevents the occurrence of severe hypoglycaemic episodes, dramatically improving the patient’s quality of life and attenuating the secondary complications of diabetes (2). However, there are too few organ donors to provide enough pancreata for even a fraction of the patients who would benefit from transplantation. This shortage has stimulated extensive research into the use of pigs as alternative islet donors. One advantage of using pigs is that they can be genetically modified to protect islet xenografts from immunological challenges including the intense innate response triggered by intraportal delivery, known as the instant blood-mediated inflammatory reaction (IBMIR) (3).

Old World non-human primates (NHP) provide the best preclinical model for xenotransplantation of adult porcine islets or neonatal islet cell clusters (NICC), because like humans they possess pre-existing antibodies to the dominant carbohydrate xenoantigen galactose-α1,3-galactose (αGal). Rhesus and cynomolgus macaques are the most widely used NHP model in islet xenotransplantation. Shin et al. achieved a median survival of 10 months for wild-type (WT) adult porcine islet xenografts in macaques (4, 5). However, a high dose of islets was required, along with a complex immunosuppressive protocol that would be unacceptable in the clinic. A more clinically applicable regimen was less effective at preventing rejection and significantly reduced the duration of insulin-independent normoglycemia (6). Graham et al. used clinically available immunosuppressants to achieve median survival of 147 days for WT adult porcine islet xenografts in macaques (7). However, recipients developed a state of systemic inflammation associated with increased incidence and severity of adverse events. Gao et al. transplanted NICC from genetically modified (GM) pigs lacking αGal (GTKO), some of which also expressed the human complement regulator CD46, into macaques treated with a clinically relevant regimen including depletional induction with anti-thymocyte globulin (ATG) (8). Median graft survival ranged from 14 to 83 days (median 40.5 days).

Baboons are arguably a more stringent preclinical model than macaques, because unlike macaques they possess IgG_3_ (9), the IgG isotype in humans that shows the strongest activation of complement and the highest degree of polymorphism. Furthermore, baboon IgG_1_, IgG_2_ and IgG_4_ show higher homology with their human homologues than do the corresponding macaque IgGs (9). Early studies in the baboon model highlighted the challenges facing pig islet xenotransplantation (10, 11). Even with profound, clinically inapplicable immunosuppression, WT adult pig islet xenografts were rejected within 28 days, with normoglycemia achieved for a maximum of only 1 day (10). More recently, we transplanted NICC from GTKO piglets expressing the human complement regulators CD55 and CD59 and human H-transferase (GTKO-CD55-CD59-HT) into non-diabetic baboons (12). The GM NICC were protected from IBMIR, but the clinical immunosuppression we used (ATG, mycophenolate mofetil and tacrolimus) was insufficient to prevent rejection within 1 month.

In this study, we aimed to extend our previous findings by achieving long-term survival and function of GTKO-CD55-CD59-HT NICC xenografts in diabetic baboons. Our simple immunosuppressive protocol consisted of depletional induction with anti-CD2, a short course of tacrolimus, and costimulation blockade-based maintenance with belatacept and anti-CD154. While two of these agents, anti-CD2 and anti-CD154, are not clinically available at this time, anti-CD2 is currently in clinical development (13) and has received FDA orphan drug approval, and safer alternatives to the thrombogenic form of anti-CD154 are under active investigation. With the combination of GM donors and our well-tolerated protocol, IBMIR was avoided, and all recipients became normoglycemic for extended periods of up to almost two years.

## Materials and Methods

### Recipient Animals and Ethics Clearances

Baboons (*Papio hamadryas*) were supplied by the Australian National Baboon Colony, National Health and Medical Research Council of Australia (NH&MRC), Sydney, Australia. All procedures were approved by both the Western Sydney and the Central Sydney Area Health Service Animal Ethics Committees and were conducted in strict compliance with State Government legislation and the NH&MRC *Principles and guidelines for the care and use of non-human primates for scientific purposes (2016)*. All protocols required comprehensive and regular external review by the Animal Research Review Panel of the New South Wales Department of Primary Industries.

### Generation of Donor Pigs

The generation of alpha1,3-galactosyltransferase knockout (GTKO) pigs (14) and CD55-CD59-HT triple-transgenic pigs that co-express human CD55, CD59 and α1,2-fucosyltransferase has been described previously (15). The three transgenes in the latter pigs, co-inherited as a single bloc, were bred onto the GTKO background. The resulting GTKO/CD55-CD59-HT pigs were mated with GTKO pigs and the offspring were genotyped as described below to identify GTKO/CD55-CD59-HT piglets, which were used as donors.

### Genotyping

GTKO/CD55-CD59-HT piglets were distinguished from GTKO piglets by multiplex Real-Time TaqMan PCR to detect the presence of the human CD55 transgene. Briefly, 10 ng of DNA extracted from ear notch biopsies was mixed with 2x TaqMan Gene Expression Master Mix, TaqMan Gene Expression Assays: CD55 (Hs00892614-g1) and βActin (Ss03376081) and assayed using 7500 Fast Real-Time PCR System as per manufacturer’s instructions (Applied Biosystems, Carlsbad, CA). Flow cytometric analysis of peripheral blood leukocytes isolated from blood collected at the time of pancreas procurement was used to confirm the absence of αGal expression and the presence of CD55 expression as described (14, 15).

### Induction of Diabetes and Peri- and Post-Transplant Monitoring

Following up to six months of training and baseline blood collection, baboons were rendered diabetic with a bolus intravenous dose of 60 mg/kg streptozotocin followed by a flush of 50 ml of normal saline. Baboons were monitored daily using blood samples obtained from a bottom prick to assess multiple daily blood sugar levels (BSLs) and (if required) ketones. At the time of a full general anaesthetic (GA), blood samples were taken from a cephalic venous canula. Bloods were collected for the first 2 weeks post-transplant *via* an indwelling central venous line. Samples and analyses included sera, plasma, full blood count, electrolytes, liver function tests, lipid studies, serum glucose, CRP, HbA1c, insulin and immunosuppression levels.

### NICC Isolation

Full GA was induced in 1-5 day old donor piglets by injection of Tiletamine (2.5 mg/kg IM), Zolazepam (2.5 mg/kg IM), and Xylazine (1.25 mg/kg IM). A midline incision was made and pancreata were dissected free from connective tissues. Once removed, pancreata were taken through a disinfection quench of povidone and washed twice in ice-cold Hank’s balanced salt solution (HBSS) (Gibco-Invitrogen, Grand Island, NY, USA) prior to isolation of NICC using a previously described technique (16). Briefly, pancreata were finely chopped and digested with 2.5 mg/ml Collagenase Type V (Sigma–Aldrich, St. Louis, MO, USA) at 37°C. Digested tissue was washed thrice in ice-cold HBSS, filtered through a 500-µm sieve and plated into Petri dishes containing Ham’s F-10 medium (Gibco-Invitrogen), 10 mM glucose, 50 mM isobutylmethylxanthine (Sigma-Aldrich), 10% pooled autologous pig sera, 2 mM L-glutamine (Invitrogen, Carlsbad, CA, USA), 10 mM nicotinamide (Sigma-Aldrich), 100 U/ml penicillin and 100 µg/ml streptomycin (Invitrogen, CA), 0.236 g/l CaCl_2_, 80 mM HEPES, and 21.3% NaHCO_3_ (Sigma-Aldrich). A full media change was performed on days 1, 3 and 5 with an additional 10 ml of media added at days 2 and 4, respectively. The cells were cultured for 6 days at 37°C in 5% CO_2_. Prior to transplantation, cultured NICC were pooled and washed twice before counting. NICC count and purity was determined using a standard counting procedure and quantitative image analysis (Aperio Positive Pixel Count Algorithm, Leica Microsystems Pty Ltd, Australia).

### NICC Transplantation

Full GA was induced in baboons by injection of 5 mg/kg ketamine, intubation with an endotracheal tube and application of 1-2% isoflurane in oxygen and air mix. Oxygen saturation, heart rate, blood pressure and partial pressures were monitored non-invasively for the duration of the operation. Prior to surgery, the abdomen was shaved and prepared with surgical antiseptic povidone. A full thickness midline incision was made from the pubis to the xiphisternum to allow exposure of the abdominal contents. The bowels were mobilised and wrapped in moist sponges and pulled aside to allow access to the vena cava. A venotomy was made in the vena cava above the iliac bifurcation for the insertion of a tunnelled central line for withdrawal of blood and infusion of immunosuppression and fluids over the first 2 weeks post transplantation. The central line was secured with a 6/0 prolene purse-string stitch to control for bleeding. The inferior mesenteric vein was mobilised and a 16G Arrow single lumen catheter (Arrow International Inc, Reading, PA, USA) was inserted, with a 6/0 prolene purse-string stitch to control for bleeding. A bolus of intravenous heparin (100 IU/kg recipient weight) was administered prior to gravity infusion of the NICC suspended in 50 ml of HBSS, followed by an additional 50 ml of HBSS. As the infusion catheter was withdrawn, the 6/0 prolene purse-string stitch was tied to prevent bleeding. Comprehensive blood samples and a full thickness liver biopsy were taken one-hour post-transplant to evaluate immediate transplantation outcomes. After this, the central line was tunnelled through the internal oblique muscle and skin and the abdominal cavity was sutured, with Marcaine local anaesthetic applied to the wound edge for pain relief. Over the duration of the operation an intravenous infusion of Hartman’s and 10% albumen was given at a rate of 10 ml/kg/hr for maintenance. The central line was secured within a protective jacket and tether system (Lomir Biomedical Inc. Quebec (Canada) that was maintained for 2 weeks for post-operative infusion of immunosuppression and fluids and collection of blood samples. Following all surgical procedures, analgesia was provided by intramuscular injection of 20 µg/kg Temgesic (buprenorphine 300 µg/ml, Indivior UK Limited, Berkshire, UK) every 6 hrs for the first 48 hrs and then as necessary.

### Liver Biopsy for Histology

Liver biopsies were taken under full GA. Briefly, a mini-laparotomy was performed above the umbilicus and a 1 cm^3^ full thickness wedge was sharp-dissected from the leading edge of the liver. The liver was sutured and diathermised to provide haemostasis and the abdomen was closed.

### Immunosuppression

**Figure 1:**
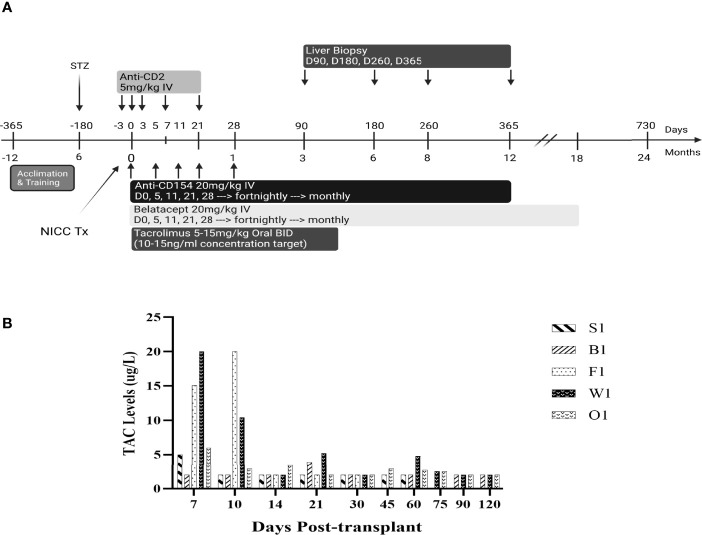
Immunosuppressive and biopsy protocol **(A)** and Tacrolimus trough levels **(B)**.

**Figure 2:**
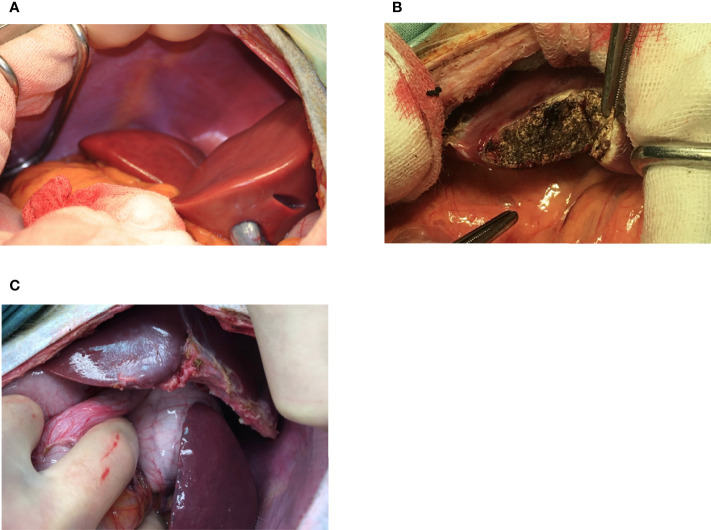
Appearance of the liver at the time of NICC infusion **(A)**, immediately after 1-hr biopsy demonstrating haemostasis following diathermising **(B)**, and 3 months post biopsy **(C)**.

All baboons received the immunosuppressive regimen shown in **Figure 1**. The anti-CD2 mAb diliximab (17) (5 mg/kg) was given intravenously on days -3, 0, 3, 7 and 21, with haematological profiles evaluated pre and post to confirm depletion of lymphocytes. Additionally, an intravenous infusion of 20 mg/kg anti-CD154 mAb 5C8 (NIH Nonhuman Primate Reagent Resource Centre) and a separate infusion of 20 mg/kg Belatacept (Nulojix, Bristol-Myers Squibb Company, New York, New York, USA) were given on days 0, 5, 11, 21, 28 and fortnightly thereafter until 6 months post-transplant, at which time dosing was changed to monthly until stopped. Recipients also received Tacrolimus (Prograf, Janssen-Cilag Pty Limited, Sydney, Australia) through the central line for the first 2 weeks, followed by twice-daily oral doses of 5-15 mg/kg up to day 120, with the dose adjusted to achieve target trough concentrations of 10-15 ug/L (**Figure 2**).

### Intravenous Glucose Tolerance Test

An intravenous glucose tolerance test (IVGTT) was performed under full GA prior to the animal being rendered diabetic, after induction of diabetes, and post-transplant at monthly intervals. An intravenous bolus of dextrose (0.5 g/kg body weight) was injected into the cephalic venous line followed by a saline flush. Blood samples were obtained before injection and at 1, 2.5, 3, 5, 7.5, 10, 15, 20, 30, 40, 45, 50, 60, 75, 90, 105 and 120 min after injection. Blood glucose concentrations were measured using a dry chemistry method on a Vitros 5.1/FS system (Ortho-Clinical Diagnostics, Rochester, NY). NHP and porcine insulin were measured using Mercodia Insulin ELISA and c-peptide concentrations were measured using a Mercodia Porcine C-peptide ELISA (Mercodia AB, Uppsala, Sweden). K values [percentage decline of natural logarithm (blood glucose)/min] were calculated with blood glucose measurements from the IVGTT.

### Oral Glucose and Arginine Tolerance Tests

An oral glucose tolerance test (OGTT) was performed by giving an oral bolus dextrose load (0.5 g/kg body weight). Blood samples were obtained before and at 10, 15, 30, 40, 60, 90, 120 and 180 min after administration of dextrose for analysis of blood glucose, porcine insulin and c-peptide. Alternatively, animals were given 5 g L-arginine hydrochloride as an IV bolus. Samples were taken at 0, 2, 4, 6, 8, 10, 15, 20, 30, 60, 90 and 120 min for analysis as above.

### Blood and Plasma Analysis

Total white cell, red cell, platelet, and differential leukocyte counts of EDTA-treated blood were obtained using a cell counter (Advia 120 Hematology System, Siemens Healthcare, Germany). Porcine C-peptide and insulin concentrations were analyzed with commercial radio-immuno-assay kits (Phadaseph RIA kit, Pharmacia Diagnostics AB, Uppsala, Sweden).

### Measurement of Anti-Pig and Non-Gal Antibodies

Anti-pig and non-Gal antibody levels in the sera of recipient baboons, pre-transplant and at selected time points post-transplant, were determined using a protocol developed by (18). Porcine aortic endothelial cells (PAEC) isolated from wild-type (WT) or GTKO pigs were used as target cells. Experiments were analysed on a FACS Canto II (BD, San Jose, CA, USA) and antibody levels were determined using FlowJo software (Tree Star, Ashland, Oregon).

### Histology and Immunohistochemical Staining

Baboon liver biopsy and endpoint samples were either mounted in OCT embedding medium (Tissue-Tek; Miles, Naperville, IL) and snap-frozen in liquid nitrogen, or placed in formalin fixative and paraffin-embedded. Sections were stained with hematoxylin and eosin or were used for immunohistochemistry. After blocking with normal horse serum, sections were stained with polyclonal guinea pig anti-swine insulin (DAKO #A0564, 1:100 – 1:200), polyclonal rabbit anti-human glucagon (DAKO #A0565, 1:200), or polyclonal rabbit anti-human somatostatin (DAKO #A0566, 1:200), with a goat anti-rabbit IgG/HRP (DAKO, Agilent Technologies, Inc, Santa Clara, CA, USA) as the secondary antibody.

Antigen retrieval was used prior to staining for chromogranin or cytokeratin (clones AE1 & AE3, BioGenex Laboratories, San Ramon, CA). All sections were counterstained with hematoxylin. Platelet deposition was detected using an anti-human platelet glycoprotein GPIIb/IIIa monoclonal antibody (R&D systems, UK), and anti-human CD3 and CD20cy antibodies (DAKO, Agilent Technologies, Inc, Santa Clara, CA, USA) were used to detect infiltrating T and B cells, respectively. Staining for αGal was performed with an anti-αGal monoclonal antibody as previously described (19).

### Image Analysis of Liver Biopsies

Stained liver biopsy sections were scanned with an Aperio Image-Scope Scanner. Sections were stained with Martius-Scarlet-Blue (MSB) trichrome for clot analysis, with chromogranin A and hematoxylin for analysis of percent NICC present, and with anti-human CD3 or CD20cy and hematoxylin for analysis of percent T and B lymphocytes present. Eight 1 mm^2^ areas were randomly selected from each image and the automated image analysis algorithm Aperio Positive Pixel Count v9 (Leica Biosystems Inc, Buffalo Grove, IL, USA) was used to quantify thrombi formation (RBC + fibrin and RBC alone). NICC were calculated as number present per section analysed; for T and B cells, the percentage of islet area staining positive was calculated.

### Statistical Analysis

The Linear Mixed Effects Model (20) was used to evaluate the hematological parameters, taking into account the variance of readings taken on the same blood donor for each variable. This was used for all full blood count results including changes to white cell count specifically lymphocyte levels. Generalized estimating equations were used to test for changes in hematological parameters over time, considering the dependence of readings taken on the same baboon. This was performed on lymphocyte counts and analysis of FACS results specifically for T and B cell count analysis (data not shown). Mean values with standard deviation are shown unless otherwise indicated. Statistical software used for data analysis was S-Plus 6 for Windows (Insightful Corporation, Seattle, WA, USA). P<0.05 was considered significant.

## Results

### Yield of NICC and Number Transplanted

Synchronized mattings were set up to produce donor piglets. We aimed to transplant between 10,000 and 50,000 IEQ per kg recipient weight; the final number was determined by the number of piglets born with the desired genotype and the yield of IEQ/g of donor pancreas weight [9,178.50 ± 4,945 (mean ± SD), range 3,621 – 15,393]. A total of 70 GTKO/CD55-CD59-HT piglets were used for six transplants into five diabetic male baboons; one animal (F1) received a second transplant to supplement an insufficient first transplant (**Table 1**). The total number of NICC transplanted ranged from 9,673 to 56,913 IEQ per kg. Viability was not significantly different between NICC batches (93.3 ± 3%), and bioindicator grafts from all batches reversed diabetes in immunodeficient mice (data not shown).

**Table 1:** Recipient and donor details along with the transplant outcomes.

Recipient Details					Donor details		Transplant Outcomes							
ID	Sex (M or F)	Age (Years)	Weight at Tx (kg)	Weight at Endpoint (Kg)	No. of Piglets used	IEQ/g donor Wt	IEQ Total Tx	IEQ per/kg Recipient Wt	Mean Fasting BSL pre STZ (Mmol/L)	Mean Fasting BSL Pre Tx (Mmol/L)	Mean daily units Insulin pre Tx	Time to come off Insulin (days)	Survival (Days)	Fate
S1	M	3.5	9.2	11	17	15,393	523,600	56,913	5.9	12.7	14	83	312	Immunosuppression withdrawn
B1	M	3.5	10.7	18.1	7	7,094	103,500	9,673	6.5	11.9	18	189	675	Immunosuppression withdrawn
F1	M	3	10.7	-	7	3,621	56,033	5,237	5.6	11.5	10	-	-	N/A first Transplant
F1 2nd Tx	M	3.5	10.8	10.8	10	14,840	201,182	18,628	-	10.2	6	110	394	Lymphoma
W1	M	2.5	5.6	6.1	10	8,988	203,840	36,400	5.2	13.5	6	20	207	Euthanased teething
O1	M	3	7.6	9.2	19	5,135	205,000	26,974	4	13.9	10	87	413	Immunosuppression withdrawn

### Immunosuppression of Recipients

Immunosuppression consisted of T cell depletion with an anti-CD2 mAb commencing at day -3, tacrolimus for the first 4 months, and maintenance with anti-CD154 and belatacept, which were progressively withdrawn in the longest-surviving animals (**Figure 1A**). Apart from a bolus of heparin (100 IU/kg) administered just prior to NICC infusion, no anticoagulant or anti-inflammatory treatment was given. All animals showed a rapid decrease in total white cell numbers associated with anti-CD2 treatment (data not shown). Tacrolimus was given intravenously *via* the central line for the first 2 weeks, then twice daily in fruit with the aim of maintaining a target trough concentration of 10-15 ng/ml. However, this was difficult to achieve, and tacrolimus levels ranged from as high as 20 ug/L whilst being given IV to <2 ug/L with oral dosing (**Figure 1B**).

### Absence of Evidence of IBMIR

**Figure 3:**
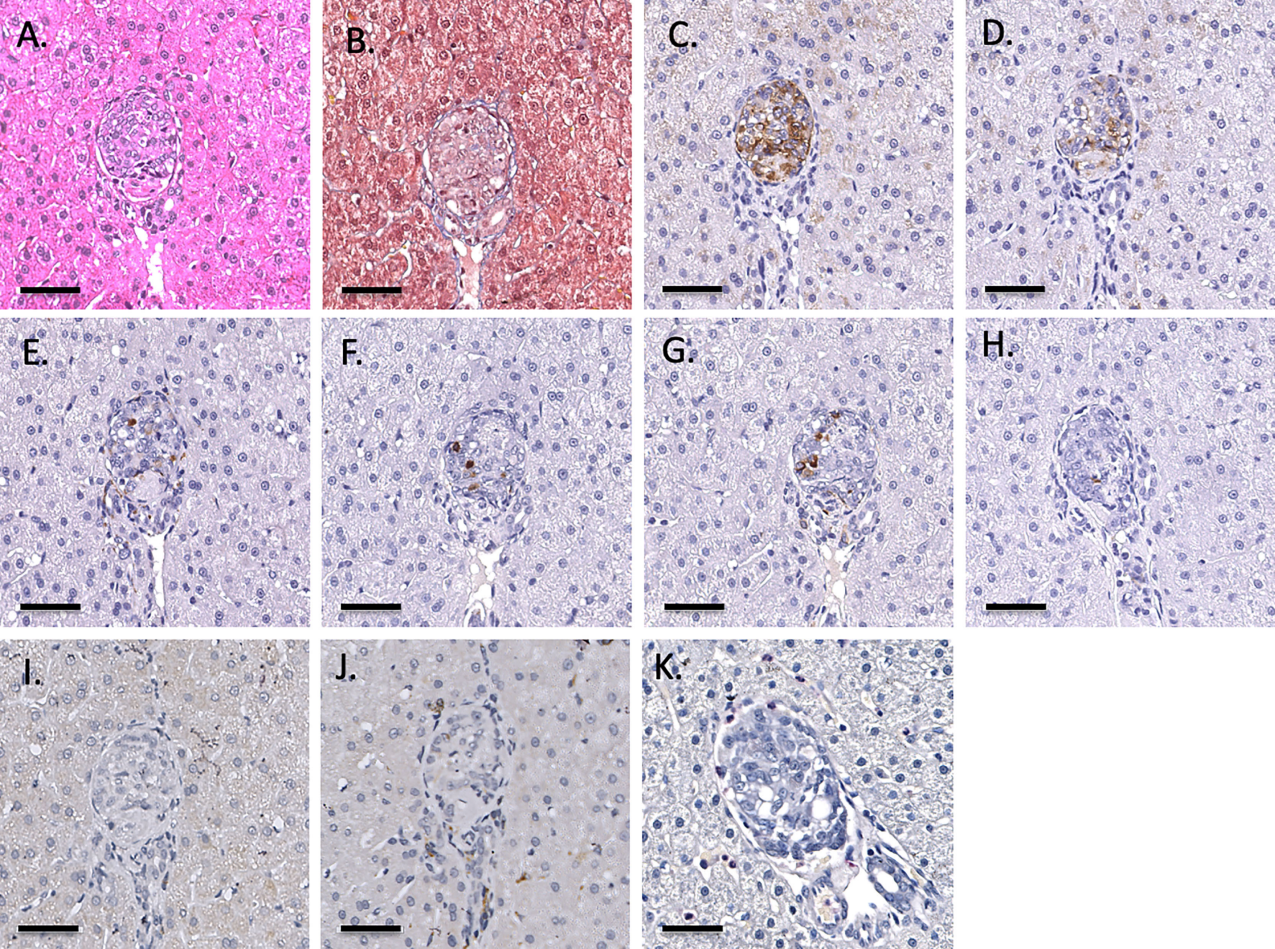
Representative 1-hr liver biopsy stained for H&E **(A)**, MSB **(B)**, CD55 **(C)**, CD59 **(D)**, chromogranin A **(E)**, insulin **(F)**, glucagon **(G)**, somatostatin **(H)**, CD20 **(I)**, CD3 **(J)** and neutrophil elastase **(K)**. Note the absence of signs of IBMIR or leukocytic infiltration. Magnification X400, indicator bar 50 um.

Infusion of NICC into the liver took a mean of 12.8 ± 4.0 min. All livers maintained a normal appearance during the infusion, with only slight blanching of the peripheral edge of the liver (**Figure 2A**). None of the livers showed evidence of vascular thrombosis. Large wedge excisional biopsies were taken at one-hour post-transplant without untoward issues (**Figure 2B**), and the liver showed excellent recovery months later (**Figure 2C**). Evaluation of one-hour biopsies showed intact NICC (**Figures 3A–B**) surrounded by normal vasculature, containing no platelets, fibrin, clot formation, or infiltration of any kind. NICC were positive for CD55 and CD59 (**Figures 3C–D**), and for chromogranin, insulin, glucagon, and somatostatin (**Figures 3E–H**), demonstrating the presence of all islet endocrine cell types. Staining for CD20, CD3 and neutrophil elastase (**Figures 3I–K**) demonstrated a lack of infiltration by B cells, T cells and neutrophils as shown in **Table 2**.

**Table 2:** An overview of histopathological outcomes in relation to the recipients clinical course.

Biopsy Identification		Immunohistochemical Staining				Genetic modification		Immune Cell Infiltration		
Recipient ID	Graft BiopsyDays post Tx	Chromogranin	Insulin	Glucagon	Somatostatin	CD55	CD59	CD3	CD20	Neutrophil Elastase
S1										
	168	++	+++	++	+	+++	+++	-	-	-
	312	++	++++	+	+	+++	+++	+++	+++	++
B1										
	406	++	++++	++	+	+++	+++	-	-	-
	675	+++	++++	++	++	+++	+++	-	-	-
F1										
	210	+	+	+	+	+	+	-	-	-
	394	++	+++	++	++	+++	+++	-	-	-
W1										
	161	+++	++++	++	+	+++	+++	-	-	-
	207	+	++	+	+	+	+	++++	++++	+++
O1										
	225	++	+++	++	+	+++	+++	-	-	-
	413	+++	++++	++	+	+++	+++	-	-	-

- means negative or none, + means minimal staining, ++ means moderate staining, +++ means heavy staining, ++++ means extremely heavy or dense infiltration.

### NICC Transplantation Restored Normoglycemia

**Figure 4:**
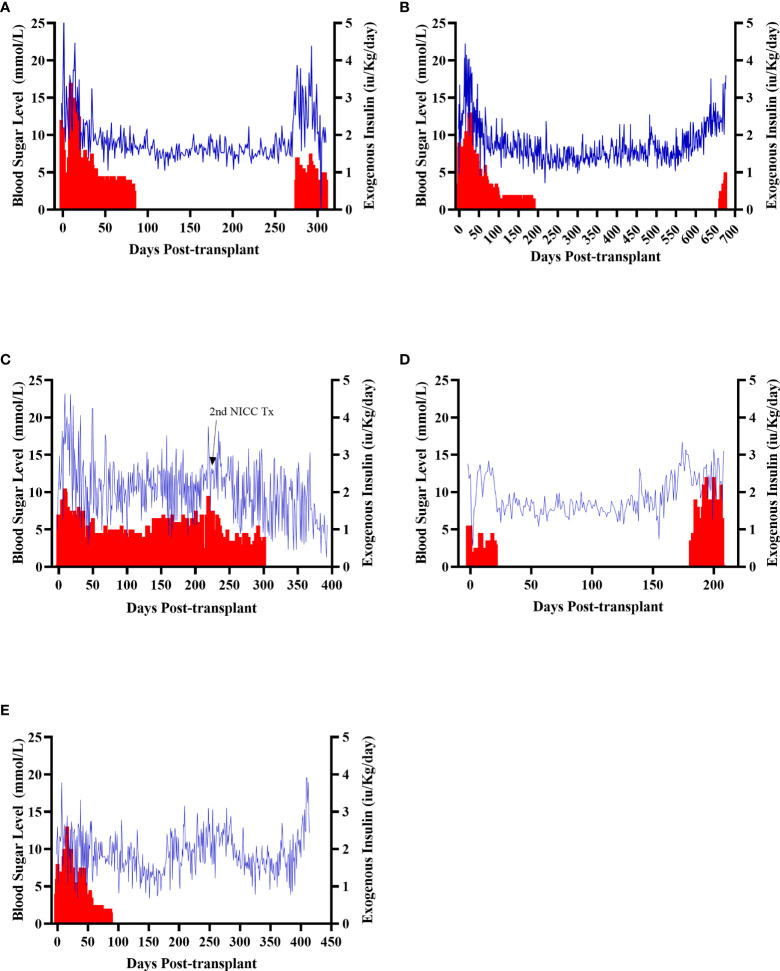
Early morning fasting blood sugar levels (blue) and daily insulin requirements (red) for all recipients. Recipients S1 **(A)**, B1 **(B)**, F1 **(C)**, W1 **(D)**, O1 **(E)**.

All recipients were profoundly diabetic prior to transplantation, with early morning fasting blood sugar levels (BSLs) regularly exceeding 15 mmol/l even with exogenous insulin treatment (**Figure 4**). After transplantation, all recipients showed a gradual decline in daily insulin requirement and a steady reduction in daily BSLs (**Figure 4**). It took between 20 and 135 days (mean 87 ± 43 days) for recipients to become insulin-independent, with insulin independence preserved for a mean of 397 ± 174 days (**Table 1**). The longest functioning graft (in recipient B1 – **Figure 4B**) survived for 675 days (22.5 months), including more than six months after withdrawal of all immunosuppression.

### Clinical Course and General Health Status

An overview of histopathological outcomes is provided in **Table 2** in relation to the recipients as outlined in their clinical course below.

**Figure 5:**
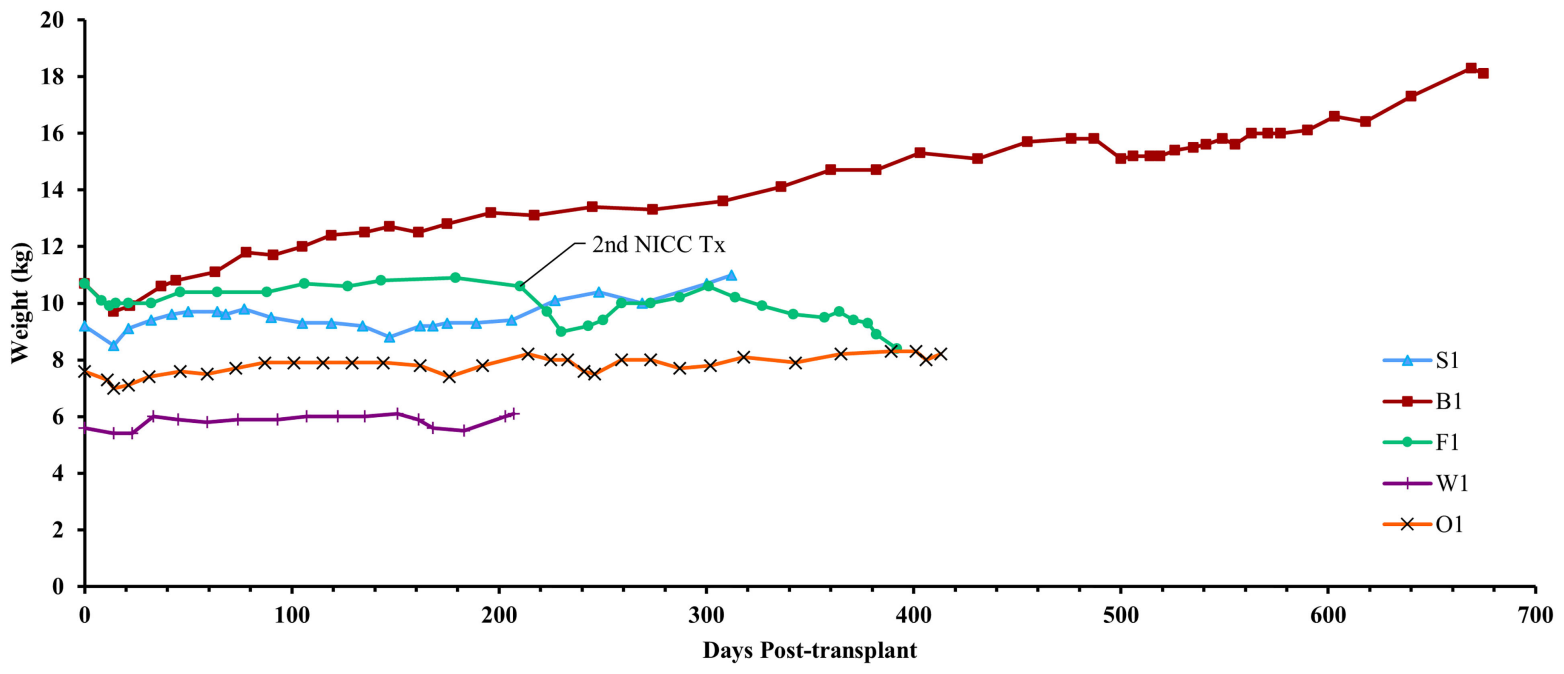
Recipient weights. After a small weight loss following surgery, all recipients gained weight over the course of the experiment. Recipient F1 initially gained weight but then lost weight after developing lymphoma.

**Figure 6:**
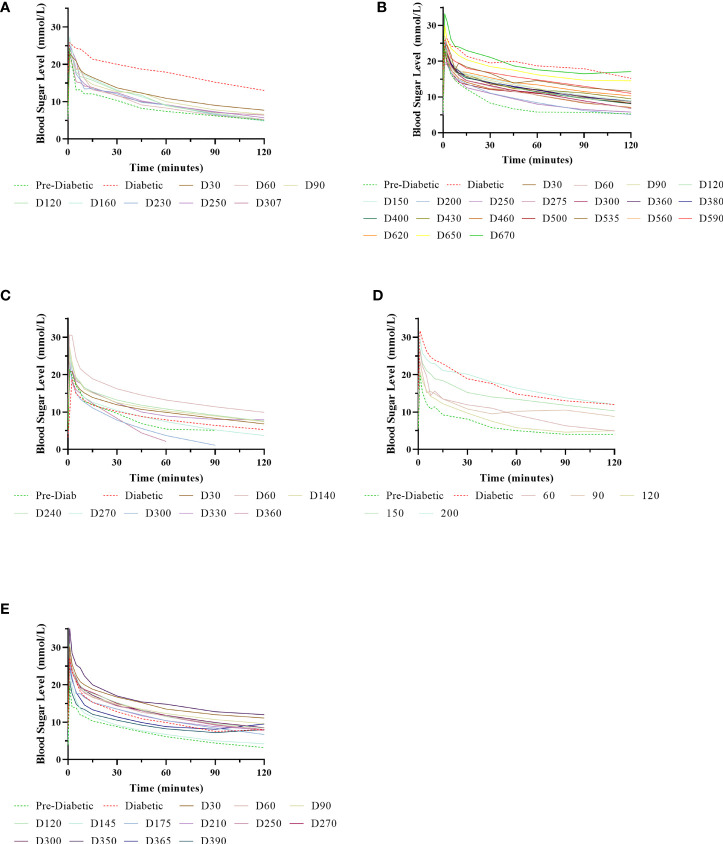
Normalisation of IVGTTs after NICC transplantation. Recipients S1 **(A)**, B1 **(B)**, F1 **(C)**, W1 **(D)**, O1 **(E)**.

**Figure 7:**
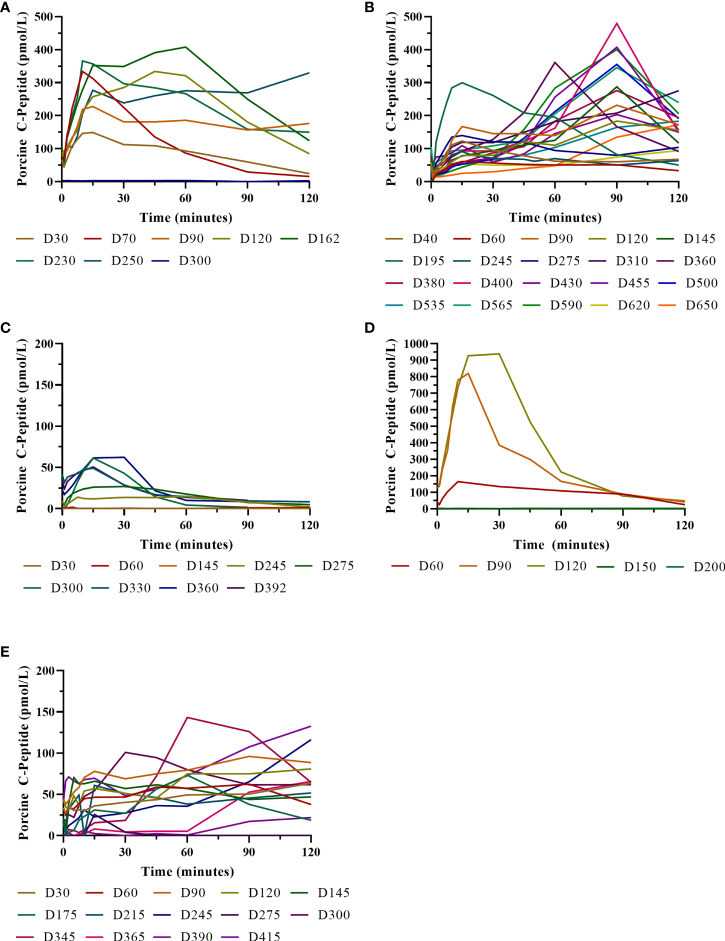
Porcine insulin and c-peptide levels during IVGTTs after NICC transplantation. Recipients S1 **(A)**, B1 **(B)**, F1 **(C)**, W1 **(D)**, O1 **(E)**.

Animal S1 had high mean daily exogenous insulin requirements utilising 14 U of long-acting insulin and was transplanted with 523,600 IEQ (56,913 IEQ/kg). Despite such high insulin requirements prior to transplant he was taken off exogenous insulin on day 83 most likely due to the larger islet cell mass transplanted (**Figure 4A**). Maintenance immunosuppression was ceased on day 227, but normoglycemia was maintained for a further 83 days until day 274 when exogenous insulin treatment was recommenced. S1 gained weight over the duration of the study as seen in **Figure 5**. S1 quickly demonstrated a return to normal BSLs in response to IVGTT (**Figure 6A**), along with corresponding porcine insulin and C-peptide levels (**Figure 7A**). S1 was euthanased on day 312 and graft pathology confirmed rejection.

Animal B1 had the highest pretransplant daily exogenous insulin requirements of 18 U of long-acting insulin and was only transplanted with 103,500 IEQ (9,673 IEQ/kg). As such it took a longer time for him to become normoglycaemic and insulin treatment was discontinued on day 189 (**Figure 4B**). After cessation of maintenance immunosuppression on day 476, B1 maintained a normal daily fasting BSL for a further 199 days. B1 demonstrated a return to normal BSLs in response to IVGTT (**Figure 6B**), along with corresponding porcine insulin and C-peptide levels (**Figure 7B**) that were maintained for over 660 days. Exogenous insulin was recommenced on day 660 due to the development of type 2 diabetes-like changes, possibly related to weight gain over the course of the experiment (from 10.7 kg to 18.1 kg, **Figure 5**). Following euthanasia on day 675, graft pathology showed enlarged insulin-positive islets.

Animal F1 was first transplanted with 56,033 IEQ (5,237 IEQ/kg), which reduced overall insulin requirements from ~16 U to ~11 U daily but was insufficient to restore normoglycemia (**Figure 4C**). F1 was treated with a second round of induction immunosuppression, and a second transplant of 201,182 IEQ (18,628 IEQ/kg) was performed 210 days after the first transplant. Exogenous insulin was ceased 91 days after the second transplant. F1 gained weight over the duration of the study as seen in **Figure 5**. F1 returned to normal BSLs in response to IVGTT (**Figure 6C**) following his second transplant and also had corresponding porcine insulin and C-peptide levels (**Figure 7C**). However, F1 developed ocular lymphoma and was euthanased with a functioning graft on ethical grounds on day 184 post second transplant (394 days post first transplant). Graft pathology showed insulin-positive islets with no signs of rejection as indicated by the absence of graft infiltration by T or B cells or neutrophils.

Animal W1 was the smallest recipient and required the lowest dose of exogenous insulin (average 6 U per day) prior to transplantation. As a result of the low insulin requirements and the fact that W1 was transplanted with 214,860 IEQ (36,400 IEQ/kg), he was taken off exogenous insulin on day 20 (**Figure 4D**). This was the earliest of all the recipients and implies that lower insulin requirements and higher numbers of NICC transplanted resulted in a faster return to normogylcaemia. W1 also exhibited the best response to IVGTT with BSLs staying tightly within the normal range for a non-diabetic baboon, and W1 had the highest porcine insulin and C-peptide levels of all recipients (**Figure 7D**) and required extra food to maintain normoglycemia. W1 gained weight over the duration of the study (**Figure 5**). Ingrowth of the adult teeth was observed at around day 200, accompanied by signs of significant inflammation including a sharp rise in WCC. This coincided with a loss of BSL control, and W1 was euthanased on day 207. Graft pathology confirmed rejection as shown in **Table 2**.

Animal O1 had a pretransplant mean daily exogenous insulin requirement of 10 U of long-acting insulin and was transplanted with 205,000 IEQ (26,974 IEQ/kg). This mid-range level of insulin requirement and islet cell mass transplanted translated to cessation of exogenous insulin on day 87 (**Figure 4E**). O1 maintained graft function for more than a year and demonstrated a return to normal BSLs in response to IVGTT (**Figure 6E**), along with corresponding porcine insulin and C-peptide levels (**Figure 7E**). He developed severe ulcerative papillomatous skin lesions at day 400 on the hands, feet, and mouth. The cause of these lesions was not definitively determined but was not related to the immunosuppression given. Despite this, O1 gained weight over the duration of the study (**Figure 5**). O1 was euthanased with a functioning graft on ethical grounds on day 413. Graft pathology demonstrated intact islets with no graft infiltration by T or B cells.

All animals were generally healthy and maintained or increased their starting body weight over the duration of the experiment (**Table 1**). There were no serious adverse events, including bacterial, fungal or viral illnesses, associated with the immunosuppressive protocol. Physical parameters including appetite, physical activity, body temperature, heart rate, and respiration were normal following transplantation (data not shown). The absence of conditions (cachexia, diarrhea, malaise, CMV reactivation) that are frequently reported in other NHP studies indicated that the immunosuppressive regimen was well tolerated and effective in suppressing xenograft rejection without impairing pathogen-specific humoral and T cell-mediated immunity. Additionally, to minimise the risk of introduction of potential pathogens to either the pig colony or the recipient animals, the pig colony was kept as a high health status herd with strict quarantine and general pathogen screening. This included screening the entire herd for PLHV2 and PCMV. Most of the breeder pigs were negative for PCMV or had exceedingly low copy numbers. The same was seen in all baboon recipients postoperatively with no (below limit of detection) or less than a mean of 100 copies per 500ng DNA.

### Graft Function as Indicated by IVGTT

IVGTTs were performed at regular intervals to evaluate graft function. All recipients entered the study with a normal BSL response during IVGTT (**Figures 6A–E**) and were rendered profoundly diabetic by streptozotocin treatment, with no native insulin or c-peptide produced after glucose stimulation. After transplantation, there was a gradual but steady return of the IVGTT profile to the pre-diabetic state, with a corresponding increase in porcine insulin and c-peptide levels (**Figures 7A–E**).

### Histopathology

**Figure 8:**
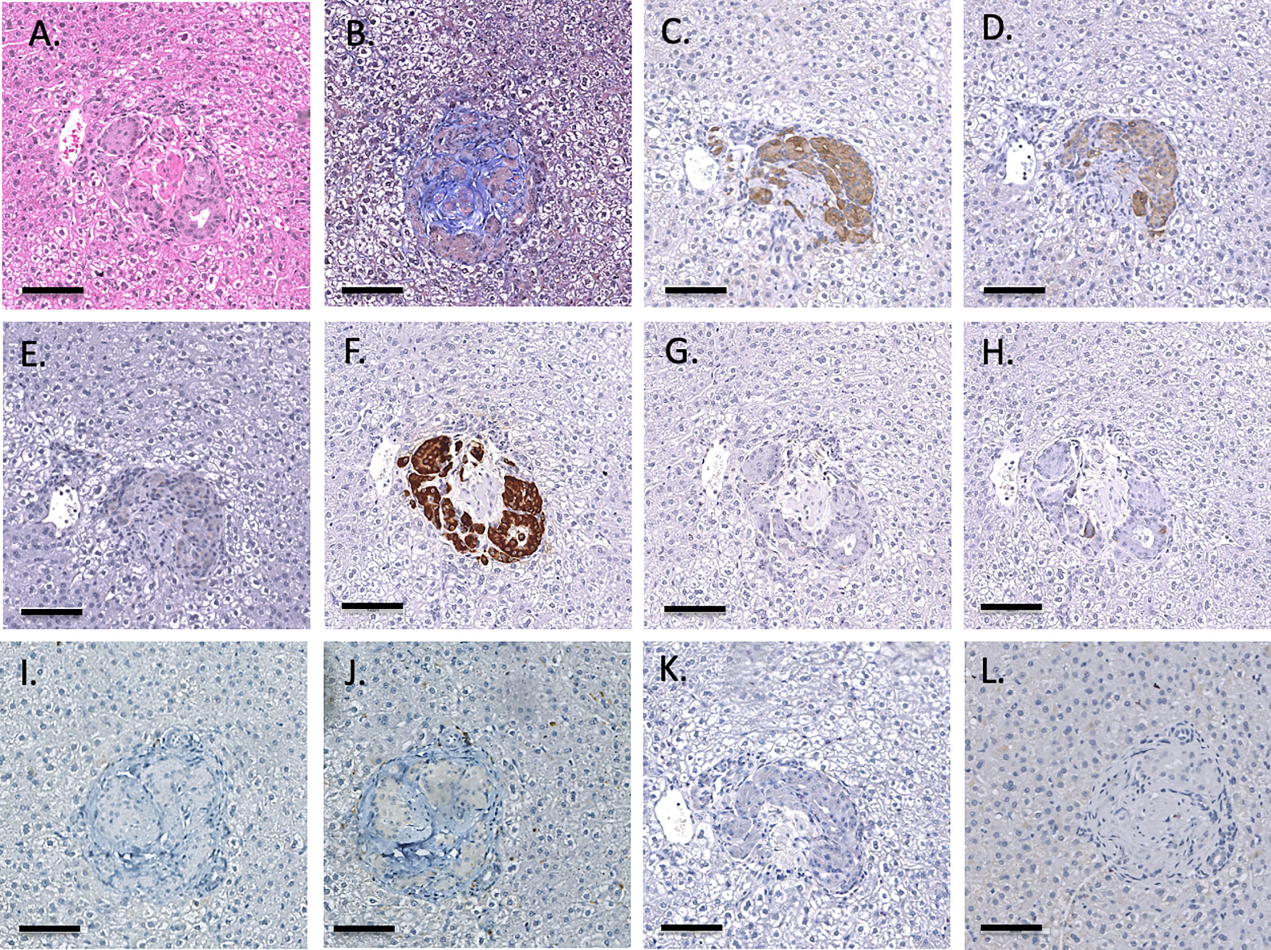
Representative 6-month liver biopsy stained for H&E **(A)**, MSB **(B)**, CD55 **(C)**, CD59 **(D)**, chromogranin A **(E)**, insulin **(F)**, glucagon **(G)**, somatostatin **(H)**, CD20 **(I)**, CD3 **(J)**, neutrophil elastase **(K)** and FoxP3 **(L)**. Magnification X200, indicator bar 100 um.

As previously described, one-hour liver biopsies showed intact NICC with little evidence of IBMIR (**Figure 3**). Subsequent scheduled biopsies performed at 6-, 8- or 12-months post-transplant demonstrated the presence of islets in all lobes of the liver biopsied, with an increase in their size and maturity over the duration of the study. A representative 6-month biopsy is shown in **Figure 8**. The islets were large and contained all islet endocrine components and remained positive for human CD55 and CD59 expression. No cellular infiltrate was observed. This has been further evaluated by individual animal NICC graft timepoint biopsy analysis as seen in **Table 2**.

### Xenoreactive Antibodies and Complement Activation

**Figure 9:**
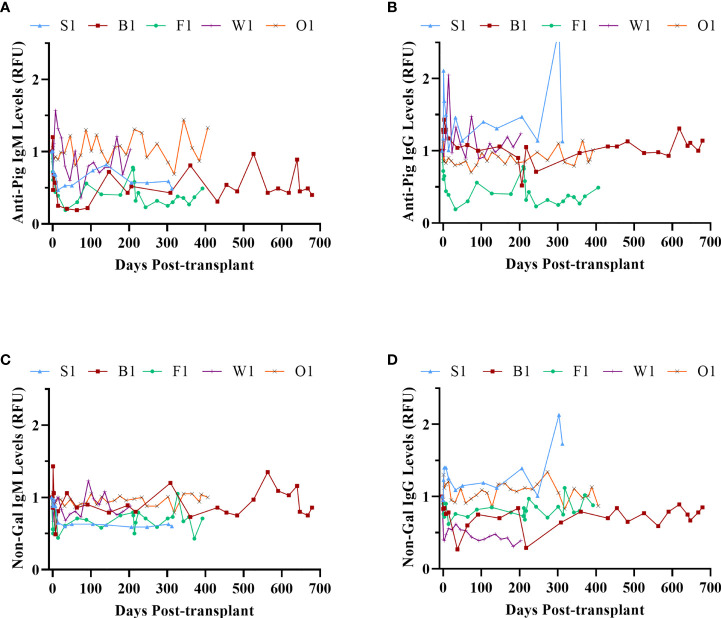
Xenoreactive antibody levels. Anti-pig IgM **(A)** and IgG **(B)**; anti-non-Gal IgM **(C)** and IgG **(D)**.

The anti-pig and non-Gal anti-pig antibody response to GTKO/CD55-CD59-HT NICC was investigated by measuring serum IgM and IgG binding to WT and GTKO pig aortic endothelial cells (PAEC) by flow cytometry. There was no significant increase in anti-pig or non-Gal IgM or IgG over the duration of the study (**Figure 9**), indicating that the immunosuppressive protocol effectively prevented the development of T-cell dependent non-Gal reactive anti-pig antibodies. Serum samples were taken from recipients at 1 hour post-transplant and analysed for complement activation by C3a ELISA. In all cases, the C3a level did not exceed baseline (data not shown).

### Other Histopathological Outcomes

**Figure 10:**
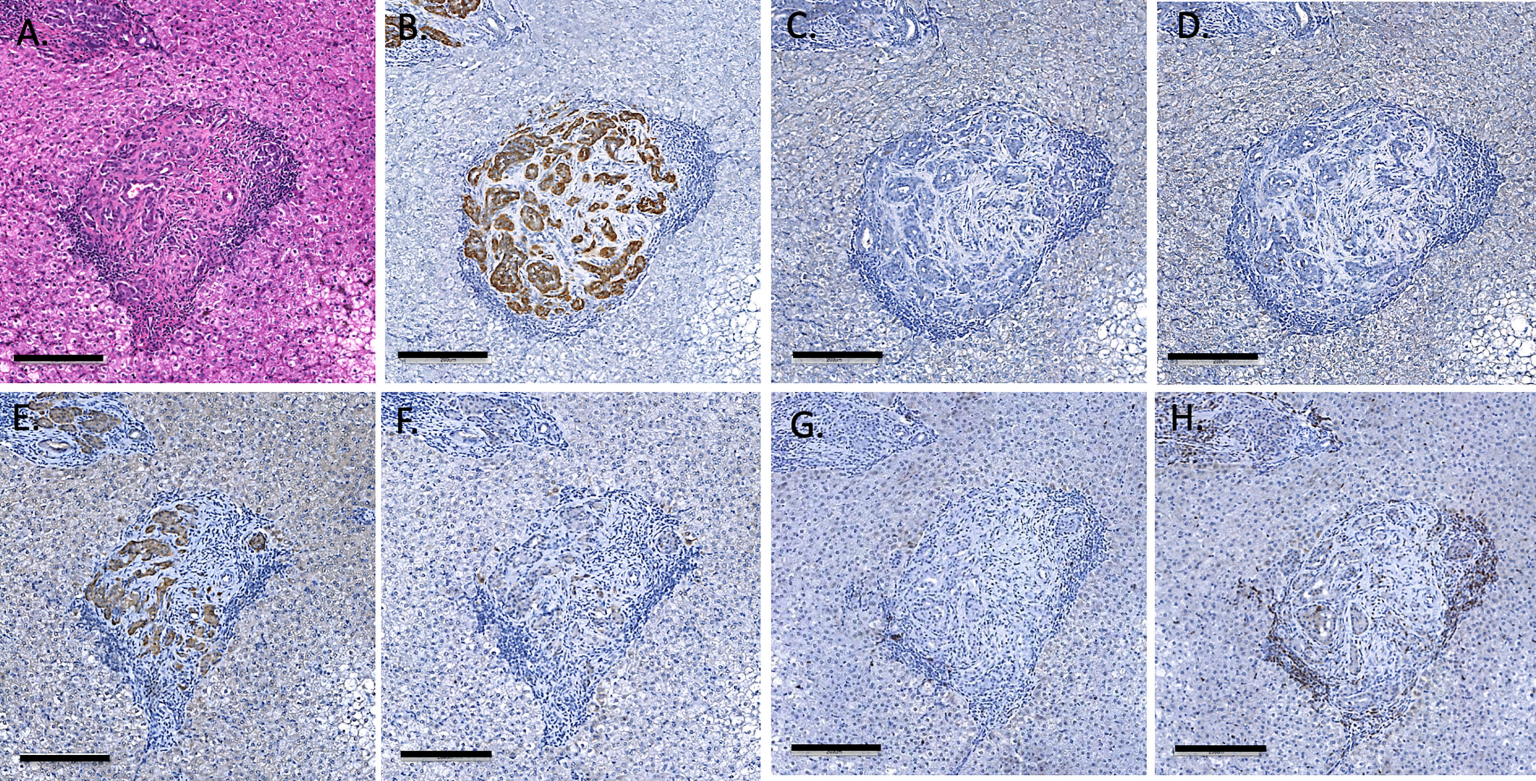
Endpoint (day 675) liver biopsy of recipient B1, showing large islets staining with H&E **(A)** insulin **(B)**, glucagon **(C)**, somatostatin **(D)**, CD55 **(E)** and CD59 **(F)**, CD20 **(G)** and CD3 **(H)**. Magnification X200, indicator bar 100 um.

All recipient animals underwent postmortem pathology to investigate any other findings such as clots, or abnormalities induced by the immunosuppression. None of the animals had any sign of micro or macro thrombi formation in any lobes of the liver or any large or small vessels, even at 2 years post-transplant (**Figure 10**). No other abnormalities were seen in any other organ or tissue examined, including the absence of any micro or macro thrombi formation in any tissues. The recipients’ native pancreata were also removed and evaluated by histology and immunohistochemistry for any sign of insulin (data not shown) indicating no recovery of native beta cells. The standard H&E sections demonstrated a lack of any islet-like structures in the native pancreas.

## Discussion

Demonstrating consistent long-term survival and function of porcine islet xenografts in preclinical NHP models is a critical prerequisite for moving forward to the clinical translation of islet xenotransplantation (21, 22). Here we report the outcome of a series of transplants of GM NICC in diabetic baboons. We used this model rather than macaques because of the closer similarity of baboons to humans in several characteristics including immune system and body temperature (9). However, baboons present specific challenges related to their size and high insulin demand; indeed, the basal daily exogenous insulin requirements post diabetes induction of the animals used in this study were almost twice those reported for macaques used in similar studies (7, 8, 23). This may help to explain the previously reported maximum survival of adult or neonatal porcine islets in baboons for only around 1 month (10–12), with normoglycemia for 1 day at most (10). Despite these challenges, we achieved, for the first time, successful long-term reversal of diabetes by transplantation of GTKO/CD55-CD59-HT NICC in five consecutive immunosuppressed baboons.

Since we had previously shown that conventional clinically based immunosuppression was unable to prevent rejection of these GM NICC (12), we used a new protocol comprising our non-activating anti-CD2 mAb (17) to deplete T cells and NK cells, tacrolimus for the first 4 months, and costimulation blockade with belatacept and anti-CD154. Gao et al. recently reported that lymphocyte depletion with ATG promoted engraftment and survival of GM NICC xenografts in macaques (8). However, anti-CD2 has several potential advantages over ATG; it favours depletion of effector T cells (24) while sparing regulatory T cells (25, 26), and avoids the adverse side effects of ATG (27). Anti-CD154 was included because of its demonstrated efficacy in NHP xenotransplantation (28). Notably, no CMV prophylaxis or treatment was given, in contrast to all other pig-to-NHP islet xenotransplantation studies (5, 7, 8). Overall, the protocol was well tolerated, with few adverse events and none of the infection issues frequently associated with transplant immunosuppression. In the macaque study by Graham et al. using clinically relevant immunosuppression including ATG induction, 50% of recipients suffered severe adverse events including post-transplant lymphoproliferative disease (PTLD) and CMV pneumonitis (7). The most severe adverse event observed in our study was the development of lymphoma in animal F1. We suspect that this was due to a combination of over-immunosuppression (F1 was subjected to two rounds of induction therapy) and a genetic predisposition to lymphoma in the closed colony from which the recipients were sourced. Of all deaths from disease or illness in the colony, 53% were diagnosed as or suspected to be lymphoma (29). The second major serious event was the development of severe ulcerative skin lesions in animal O1. Although the cause of these lesions could not be determined, there was no evidence linking them to the immunosuppressive regimen.

Additional issues in previous NHP studies included weight loss and various concerns of general recipient health and wellbeing. Several macaque studies encountered multiple issues in animals treated with various combinations of immunosuppression including clinically relevant induction with anti-thymocyte globulin (ATG). These protocols were almost always associated with weight loss which was commonly also associated with cachexia and diarrhea, and in some studies occurred in up to 70% of recipients (5, 7). In our study importantly we did not observe weight loss, conversely, we saw a steady increase in weight over the duration of the entire study period, with the exception of animal F1, which initially gained weight but then started to lose weight after developing lymphoma. All other animals showed significant weight gain overall and one animal increased 70% from his starting weight.

In this study the dose of GM NICC required to reverse diabetes long-term was as low as 10,000 IEQ/kg. This contrasts with the outcome reported for NICC xenografts of 39,000 – 115,000 IEQ/kg, which did not engender insulin independence and only survived for a maximum of 83 days (8). Remarkably, the baboon in our study receiving the lowest islet dose (B1) remained normoglycemic despite gaining weight from 10.7 kg to 18.1 kg. This suggests that neonatal porcine islets protected by appropriate genetic modifications and effective immunosuppression can enlarge to keep pace with the requirements of the recipient. Furthermore, the grafts in two recipients (S1 and B1) continued to function for up to six months after all immunosuppression was withdrawn, with no signs of rejection including an antibody response. This suggests the development of a state of operational tolerance. Although rejection eventually ensued in S1, the liver of B1 showed the presence of enlarged infiltrate-free, insulin-positive islets even after blood glucose control was lost, indicative of islet exhaustion rather than rejection.

One limitation of our study was the use of anti-CD154, which is currently not available for clinical use due to the risk of potentially fatal thromboembolic complications (30). However, forms of anti-CD154 with modified Fc domains designed to avoid these complications are being investigated in NHP models of allotransplantation (31) and xenotransplantation (32) and have reached the stage of clinical trials (NCT03605927; NCT04322149). Another option may be to block the other side of the anti-CD40/anti-CD154 interaction. Anti-CD40 has been used as a substitute for anti-CD154 in xenotransplantation with some success (32, 33).

Based on the successful outcomes of this study, we propose to move this work forward by developing tolerising protocols using Treg therapy and/or further genetic manipulation of the donor pig to suppress the T cell-mediated response, for example by the transgenic, graft-mediated local expression of immunosuppressive agents such as CTLA4-Ig (34) or anti-CD2 (14, 17, 35). The inherent benefits of producing tolerance of the graft need to be utilized further to move islet xenotransplantation to the clinic.

## Data Availability Statement

The raw data supporting the conclusions of this article will be made available by the authors, without undue reservation.

## Ethics Statement

The animal study was reviewed and approved by Western Sydney Local Health District Animal Ethics Committee.

## Author Contributions

Conceptualization: WH and PC; Methodology: WH; Performance of work: WH, ES, YC, HBu, JH, HBa, MH, and PC; Data Curation: WH, ES, and YC; Writing – Original Draft: WH and PC; Review & Editing: All authors; Visualization: WH, ES, and YC; Supervision: WH and PC; Project Administration: WH; Funding Acquisition: PC and WH. All authors contributed to the article and approved the submitted version.

## Funding

This work was supported by grants from the National Health and Medical Research Council of Australia (Project Grants 1061868 and 1156889; NHMRC/JDRF Program Grant 447718) and the Juvenile Diabetes Research Foundation (3-SRA-2017-366-S-B).

## Conflict of Interest

The authors declare that the research was conducted in the absence of any commercial or financial relationships that could be construed as a potential conflict of interest.

## Publisher’s Note

All claims expressed in this article are solely those of the authors and do not necessarily represent those of their affiliated organizations, or those of the publisher, the editors and the reviewers. Any product that may be evaluated in this article, or claim that may be made by its manufacturer, is not guaranteed or endorsed by the publisher.

## Abbreviations

αGal, galactose-α1, 3-galactose; ATG, anti-thymocyte globulin; GA, general anaesthetic; GM, genetically modified; GTKO, *GGTA1* gene knockout; GTKO/CD55-CD59-HT, GTKO pigs expressing human CD55, CD59 and α1,2-fucosyltransferase (H-transferase, HT); HAR, hyperacute rejection; IBMIR, instant blood-mediated inflammatory reaction; IEQ, islet equivalents; NICC, neonatal islet cell clusters; NHP, non-human primates; PAEC, porcine aortic endothelial cells; PPP, platelet-poor plasma; SD, standard deviation; mAb, monoclonal antibody; WT, wild-type.
